# Pupil Dilations Reflect Why Rembrandt Biased Female Portraits Leftward and Males Rightward

**DOI:** 10.3389/fnhum.2013.00938

**Published:** 2014-01-13

**Authors:** James A. Schirillo

**Affiliations:** ^1^Department of Psychology, Wake Forest University, Winston-Salem, NC, USA

**Keywords:** hemispheric laterality, pupil size, face perception, emotion, esthetic judgments

## Abstract

Portrait painters are experts at examining faces and since emotional content may be expressed differently on each side of the face, consider that Rembrandt biased his male portraits to show their right-cheek more often and female portraits to show their left-cheek more often. This raises questions regarding the emotional significance of such biased positions. I presented rightward and leftward facing male and female portraits. I measured observers’ pupil size while asking observers to report how (dis)pleasing they found each image. This was a methodological improvement over the type of research initially done by Eckhard Hess who claimed that pupils dilate to pleasant images and constrict to unpleasant images. His work was confounded since his images’ luminances and contrasts across conditions were inconsistent potentially affecting pupil size. To overcome this limitation I presented rightward or leftward facing male and female portraits by Rembrandt to observers in either their original or mirror-reversed position. I found that in viewing male portraits pupil diameter was a function of arousal. That is, larger pupil diameter occurred for images rated both low and high in pleasantness. This was not the case with female portraits. I discuss these findings in regard to the perceived dominance of males and how emotional expressions may be driven by hemispheric laterality.

## Introduction

Portraitures have been shown to exhibit a leftward bias, where the left check is exposed more often than the right. This occurs more often in female than male portraits which may be due to a desire to portray female’s more emotive left-side. Hemispheric lateralization may also play a role by projecting negative emotions to the left-side of the face and positive emotions to the right-side of the face. This distinction will be addressed. Given that conscious judgments of pleasingness are beset with problems, it is important to also use an unconscious measure such as pupil size. Hess (1965, 1972) claimed that pupils enlarge when viewing pleasant images and constrict when viewing unpleasant images. Yet since pupil size also covaries with luminance, a novel technique was employed to measure pupil size in response to portraits and their mirror image. I found that Hess was incorrect to focus on valence and should have emphasized arousal instead. This finding was evident in male but not female portraits, which may be due to the dominance exhibited in male portraitures.

### Portraiture’s leftward bias

Portraits often expose more of one side of their face than the other side (McManus and Humphrey, 1973; Grüsser et al., 1988). For example, in a study of 1,474 Western European portraits created from the fourteenth to the twentieth century, 891 posers (~60%) exposed more of their left-cheek, whereas 583 (~40%) exposed more of their right-check (McManus and Humphrey, 1973). This left-cheek asymmetry is stronger for women portraits (Gordon, 1974; Grüsser et al., 1988; Conesa et al., 1995). That is, ~68% of women’s portraits, but only 56% of male portraits have a leftward bias (McManus and Humphrey, 1973).

This gender difference has had several explanations (Lindell, 2013). One promising interpretation suggests that the leftward bias results from the poser’s preference to portray the left-side of the face’s emotional qualities (Nicholls et al., 1999). Nicholls et al., 1999, p. 665) instructed participants to pose for a portrait to either “put as much real emotion and passion into a portrait as you can” or “to avoid depicting any emotion at all.” In the first case, participants were more likely to turn their left-cheek toward a camera during a picture-taking session, whereas in the second case participants were more likely to turn their right-cheek toward the camera. Likewise, multiple studies report that the left-side of the face is more intense in exhibiting voluntary emotional expression, especially for women (Sackeim and Gur, 1978; Sackeim et al., 1978; Borod and Caron, 1980; Borod et al., 1988; Nicholls et al., 2000). For example, Nicholls et al. (2002a) demonstrated that people who are more emotionally expressive are more likely to pose for a portrait offering the left-cheek; the paper argues that as females score higher on measures of emotional expressivity, they are more likely to pose offering the left-cheek. Nicholls et al. (2002b) then demonstrated that viewers perceive images of models offering the left-cheek as more emotionally expressive. Lindell (2013) offers a review of this literature. These findings are consistent with the idea that facial expressions are related to cerebral hemispheric laterality, and that the right brain hemisphere is dominant in processing and also displaying emotional expressions (to the left-side of the face) (Bryden and Ley, 1983).

Given these findings, males may not want to portray their emotive left-side as much as females (or by the behest of the artist). Likewise, artists may prefer to portray women as being more emotive than men, thereby exposing their left-cheek more often. These notions are supported by Grüsser et al. (1988), who examined fifteenth to twentieth century portraits and found a left-cheeked bias, which was always stronger for female than for male portraits. Thus, the current study examines the bias a portrait conveys, not the viewer’s preference for a right or left-side of an image. This tests the sitter’s hemispheric asymmetry (contralateral control of facial musculature) rather than the perceiver will produce differences in valence or arousal.

Alternatively, a valence hypothesis suggests that since each cerebral hemisphere controls predominantly the musculature on the lower two-thirds of the contralateral side of the face (Brodal, 1965) each side of the face portrays different emotive qualities. So, positive emotions should be more prevalent on the right-side of the face (since it is governed by the left cerebral hemisphere) and negative emotional expressions should be more prevalent on the left-side of the face (since it is governed by the right cerebral hemisphere) (Rossi and Rosadini, 1967; Gainotti, 1969, 1972; Ahern and Schwartz, 1979; Schwartz et al., 1979; Sackeim et al., 1982; Fridlund and Izard, 1983; Natale et al., 1983; Sackeim and Gur, 1983; Davidson, 1984; Silberman and Weingartner, 1986; Schiff and Lamon, 1989; Mandal et al., 1991; Borod et al., 1997; Jasari et al., 2000; for a literature review see Powell and Schirillo, 2009). This makes it peculiar to portray the left-cheek more often. Logically it may follow that the valence hypothesis is less compelling than the lateralized one.

**Figure 1:**
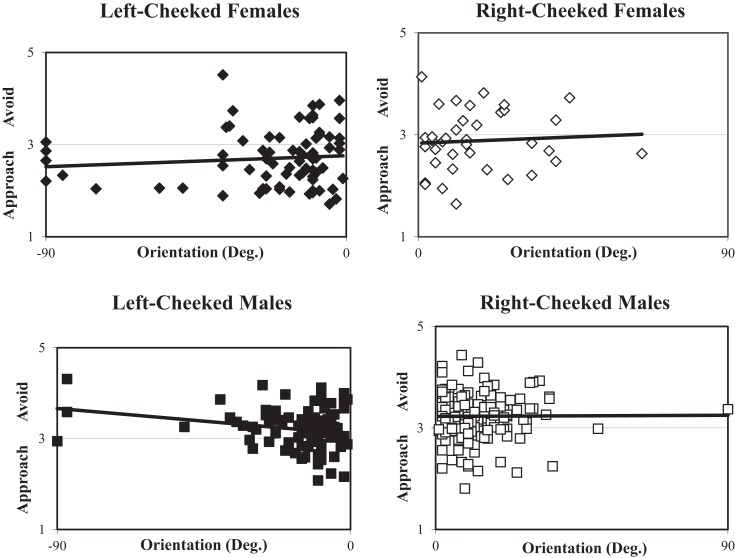
**Scatterplots of the distribution of each of 373 Rembrandt portrait angles as a function of ratings of approach/avoidance ratings (collapsed over 73 subjects’ ratings)**. Upper left (closed squares) – shows left-cheeked males (−90° to −1° orientation), upper right (open squares) – shows right-cheeked males (+1° to +90° orientation), lower left (closed diamonds) – shows left-cheeked females (−90° to −1° orientation), lower right (open diamonds) – shows right-cheeked females (+1° to +90° orientation). Linear regressions are plotted for each graph. Here 2 indicates a rating of “mildly approach,” 3 indicates “neutral,” and 4 indicates “mildly avoid.”

Thus, the field of lateralized portraiture has sought to determine whether one side of the face is more pleasant than the other. Schirillo and Fox (2006) (Figure 1) showed observers all 373 of Rembrandt’s portraits and found that left-cheeked females were assessed as more approachable than right-cheeked females portraits while males portraits (for both sides of the face) were assessed as preferably avoided. Thus, observers were more likely overall to want to approach female rather than male Rembrandt portraits. Unfortunately, this study did not use mirror-reversed images so it could not determine overall effect of cheek.

### Conscious emotional judgments measured using an unconscious pupil size measure

Research investigating the interaction of lateralized portraiture and pleasantness ratings is beset with problems (Rinn, 1984). For example, the most popular methodology is to obtain an observer’s subjective impression of the stimuli such as degree of liking (Russell and George, 1990). This method may cause observers to use separate, immeasurable, criteria in making judgments (e.g., one observer may use an image’s contrast, whereas another may use facial features, such as eyebrows). This obfuscates linking esthetic judgments to cerebral laterality. Thus, it makes sense to try to understand portrait laterality to what may be a correlate to automatic affective reactions.

Pupil size is one previously measured unconscious indicator of affective processing (Hess and Polt, 1960; Hess, 1965, 1972; Janisse, 1974; Loewenfeld, 1999). Hess (1965, 1972), attempted to transform the field of esthetics by claiming that pupils enlarge when viewing pleasant images and constrict when viewing unpleasant images (see also Hess and Polt, 1960; Hess et al., 1965; Simms, 1967; Fitzgerald, 1968; Goldwater, 1972). However, pupil size also varies with luminance (Loewenfeld, 1966, 1999; Woodmansee, 1966; Miller, 1967; Kohn and Clynes, 1969; Goldwater, 1972; Janisse, 1973, 1974; Loftus, 1985; Mannan et al., 1995; Locher, 1996), and since Hess compared different images with different intensities and contrast levels (e.g., a snake versus a naked women), his work was confounded.

### A novel methodology

Since I used original and mirror-reversed portraits, I was also able to explore the hemispheric laterality of emotional expression. Rembrandt may have turned his subject’s faces sideways to display specific emotional content of their facial musculature. If this is the case, self-reports regarding dominance especially of male subjects should show up as emotional responses which might be quantified by pupil size relationships. It was found that Hess was incorrect to focus on valence. Instead, he should have focused on arousal, since it was found that arousal, not valence, drives pupil diameter for male portraits. That is pupils dilated for males that were rated both most pleasant and most unpleasant (Powell and Schirillo, 2009). Thus, verbal ratings of pleasantness are a self-report measure which relate to an unconscious indicator of pleasantness (i.e., pupil diameter) as suggested by Hess and others (Hess and Polt, 1960; Hess, 1965, 1972; Hess et al., 1965).

### Dominance, valence, and arousal

Dominance (typically associated with positive, e.g., self-assurance, arrogance, and feeling bold or triumphant) or negative affective states (e.g., hostility, irritability, and anger) (Demaree et al., 2005) can lead to larger pupil size (Darwin, 1872). These arousal differences should only be present in viewing male portraits since they accentuate dominance (Dunbar and Burgoon, 2005). This is emphasized in images where each side of the face reflects different emotional expressions a concept first posited by Darwin (1872), especially as it relates to dominance.

The use of Rembrandt portraits allowed for the exploration of esthetic judgments of a famous artist while investigations of Rembrandt’s work in the context of hemispheric laterality provide also examining potential differences in facial emotion expression. First, using artwork should elicit stronger esthetic reactions than photographs of faces. Second, prior preliminary evidence that perceived dominance is greater when viewing his right-cheeked male portraits (Schirillo, 2000). It is possible that this is the reason for their prevalence; however due to the correlational nature of the data it may also be that Rembrandt may have chosen to selectively portray the right-cheeks of more dominant males. Thus, I attempt to show how hemispheric asymmetries may regulate displays of facial emotion which are reflected by an observer’s esthetic judgment of a portrait. It is interesting that Schirillo’s (2000) study of “social appealingness” of male and female right and left-cheeked portraits differs from Schirillo and Fox’s (2006) study of approach/avoidance of the same? Seemingly, “social appeal” differs from the desire to approach or avoid. Further work on this discrepancy is needed.

Given that verbal ratings of pleasingness are a self-report measure, it is of interest to determine their relationship with an unconscious indicator of pleasingness (i.e., pupil diameter) as suggested by Hess and others, and how this in turn might be related to the emotional content of facial musculature. If self-report interpretations drive assumptions regarding dominance, they may show up in the portraits’ emotional qualities which are reflected in pupil size relationships. However, it may be that pupil diameter is more related to arousal rather than pleasingness. The current study will help clarify this dependent variable.

In an earlier study of Rembrandt portraits (Schirillo, 2000) a factor analysis revealed that females with their left-cheek exposed were judged to be much less socially appealing than less commonly painted right-cheeked females. Conversely, the more commonly painted right-cheeked males were judged to be more socially appealing than either left-cheeked males or females facing either direction. It was hypothesized that hemispheric asymmetries regulating emotional facial displays of approach and avoidance influenced the side of the face Rembrandt’s models exposed due to prevailing social norms. Thus, females would be considered more appealing than males and left facing males would be considered the least appealing. A second experiment had different subjects judge a different collection of 40 portraits by Rembrandt and their mirror images. Portraits were matched for valence, arousal and dominance by a second set of 20 subjects. I hypothesized that mirror-reversed images would produce the same pattern of results as their original orientation counterparts. I also hypothesized that hemispheric asymmetries that specify the emotional expression on each side of the face will account for the obtained results, that is, original left-cheeked males will be preferred, due to their perceived dominance while there will be no difference in female portraits.

## Materials and Methods

### Subjects

Forty right-handed observers (20 males; ages 18–23) with normal or corrected-to-normal vision (but no eyeglasses) from the introductory psychology research pool at Wake Forest University participated in the study. Handedness was determined using Annett’s Peg-Moving task as right-minus-left latency (peg-moving speed) (Annett and Kilshaw, 1983). The study was performed in accordance with the ethical standards of the Declaration of Helsinki.

### Stimuli

**Figure 2:**
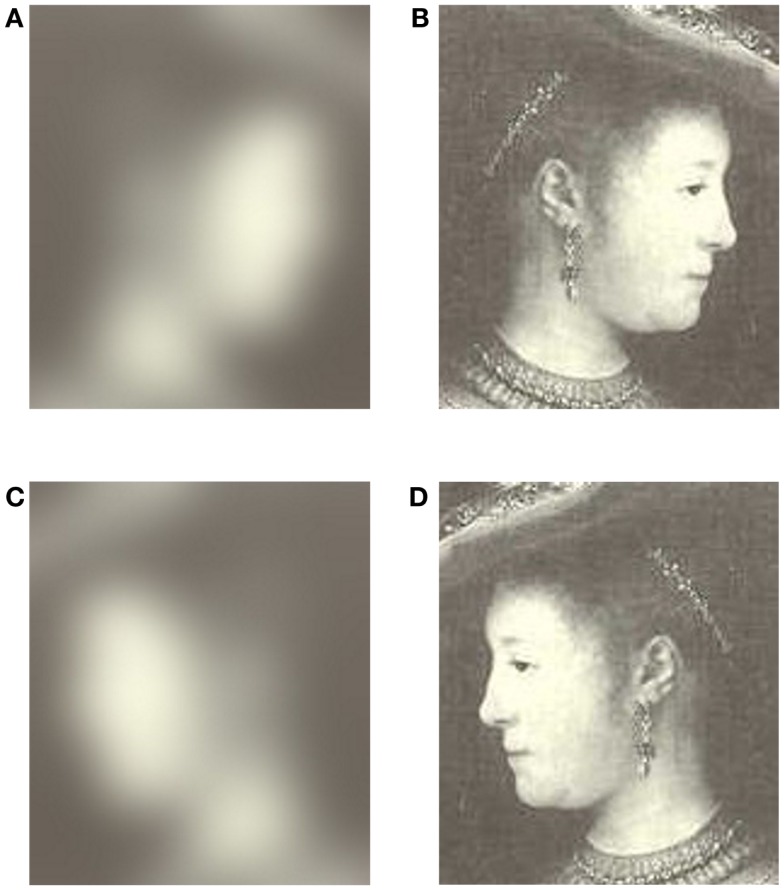
**(A)** Blurred original orientation, **(B)** original orientation of a left-cheeked female, **(C)** blurred mirror-reverse, and **(D)** mirror-reverse orientation (A woman in fanciful costume). Baltimore, The Walters Art Gallery; Br. 386). Copyright 1969 Phaidon Press Ltd. Rembrandt, *The Complete Edition of Paintings*. A. Bredius, revised by H. Gerson.

Forty black and white images taken from oil paintings were chosen from a collection of 373 portraits painted by Rembrandt. The specific portraits chosen represent his most rightward and leftward facing portraits (Schirillo and Fox, 2006). Grayscale images were used instead of colored images because color can lead to changes in pupil diameter (Miller, 1967; Kohn and Clynes, 1969). Ten were right-cheeked males (none were self-portraits of Rembrandt), 10 were left-cheeked males, 10 were right-cheeked females, and 10 were left-cheeked females (Lists Painting Names in Appendix). Next, these images were used to produce 40 mirror-reversed images using PhotoShop IBM. The portraits were only of busts. Each portrait was scanned into PhotoShop and was projected to each observer individually using an IBM CRT computer monitor using Microsoft PowerPoint. Viewing distance was 24″ making the image size range from 11.7°(height) × 8.5°(width) to 11.8° × 12.4°. The observers close distance to the screen limited their ability to spend considerable time viewing off-screen. To verify this notion, since the head-mounted Applied Science Laboratories (ASL; series 6000) eye-tracker could also measure eye position, I determined that observers were only off-screen ~3% of their total viewing time. My data also showed that time with no record (due to eye closure) was minimal. In addition, 80 blurred images were created (40 from original and 40 from mirror-reversed images) in PhotoShop using a Gaussian blur function (See Figure 2). There was no ambient lightning in the experimental chamber, in that it was a room without windows. Since the door was closed, the only light available came directly from the computer screen that showed the images.

The eye tracking device was used to determine the pupil size of the left-eye. The right-eye pupil size was not measured because pupil size is believed to be conjugate across the two eyes (Loewenfeld, 1999). Pupil diameter was recorded automatically every 17 ms for each entire 15 s trial. The average size across the entire 15 s viewing period was computed minus any time the pupil computation was off-line (due to blinks, etc.). Given that the ASL eye-tracker stops recording when the eye closes more than 50% (assumedly due to blinks or partial eye closure) I have no record of this data.

Instead of using linear interpolation to estimate the pupil size during this off-line period (Steinhauer et al., 2004) I felt it best to simply eliminate these segments from my dataset since blinks can alter pupil size (Nakayama, 2006). Given that the ASL eye-tracker is fixed to the head, head-movements did not alter pupil diameter recordings or result in loss of tracking. Thus, other than eye-blink time I did not remove any artifacts from the data. The remaining data was imported into Excel to do the data cleaning which was then converted to SPSS to do the statistical analysis. Average pupil size was calculated for each image across the observation period which excluded instances where the observer blinked or had partial eye lid closures (since when the eye closes the ASL machine cannot record any pupil size). Observers used a chinrest to ensure a fixed 24″ distance between themselves and the screen to retain a constant depth of field across the images (Simms, 1967).

To circumvent Hess’ luminance and contrast confounds, I had observers view left- and right-cheeked portraits, and their mirror images (e.g., see Figures 2B,D), while they determined the esthetic pleasantness of each face. Simultaneously, I monitored their pupil size allowing for a correlate between portrait pleasantness and pupil size. Since original and mirror images have the same luminance profiles, and I only compared pupil size and pleasantness ratings across matched pairs of faces, Hess’ confounds were eliminated.

Since Woodmansee (1966, p. 133) found “significant pupillary constriction with shifts in gaze from darker to brighter areas of the picture,” I, like him, presented a blurred image prior to its clear image to minimize changes in pupil size. For example, Figure 2 shows images in their original and mirror-reverse orientation (Figures 2B,D) along with their corresponding preceding blurred images (Figures 2A,C). These images are significantly blurred, so that facial pleasantness cannot be extracted from the blurred images (Bachman, 2007).

Observers viewed 40 images in their original posed orientation, and in their mirror-reversed orientation (resulting in 80 images in total). Since I only compared pupil size across original versus mirror-reversed images, my within-subject’s design eliminates potential confounding factors such as age and medication. Right-cheeked mirror-reversed images are portraits that originally faced rightward but due to reversal appeared to be of original left-cheeked images. Likewise, left-cheek mirror-reversed images appeared to be of original right-cheek portraits. Images were randomized, and presented to each observer in random order. Each of the 80 images was viewed for 15 s and was preceded by a blurred version of the image for 15 s. Fifteen seconds was decided upon because of three previous findings. First, Smith and Smith (2001) found that art viewers examined The Metropolitan Museum of Art paintings for a median of 17 s. Second, Aboyoun and Dabbs (1998) showed that pupil size rapidly decreases upon image presentation, which then recovers to either baseline or above baseline levels. Consequently, pupil size must be measured for at least several seconds to overcome this initial depression. Lastly, Richer et al. (1983) found that pupil size increases begin about 1.5 s before stimulus presentation and peak around a second after presentation. As a result, I gave observers more time than needed to generate an entire response to an image. Observers’ pupil size was measured during each non-blurred image presentation while they contemplated how pleasant they found the non-blurred images. Observers were instructed to think about the esthetic pleasingness of each image for the entire 15 s it was shown and then report their judgment after the image was removed. This occurred during the presentation of the subsequent blurry image.

The rationale for presenting a blurred image of a given portrait prior to presenting that portrait was to avoid the following confound prevalent in the pupillometry literature. That is, if a constant blank gray screen was used as a baseline the subsequent test-image would produce the following effect (See Figure 2 taken from Bradley et al., 2008). That is, the most important natural function of the pupil is to dynamically respond to changes in environmental illumination with an initial constriction (i.e., the light reflex) that is related to stimulus luminosity (Beatty and Lucero-Wagoner, 2000). Thus, if a constant blank gray screen were used as a baseline the brighter images would produce a larger constriction than the dimmer images. This effect takes up to 6 s before reaching a plateau. To circumvent this effect I choose to first present for 15 s a blurred image of the subsequent test-portrait. This does two things. First, it makes the large constriction (i.e., light reflex) occur during the blurred image rather than during the test-image. By the end of the 15 s of viewing the blurred image the pupil has adjusted to the light level of the image that will subsequently be presented. Given this very long duration the pupil will no longer carry-over any information from the previous clear portrait. This is because the pupil is reflexive and does not contain a memory loop so by the end of presenting a blurred image there are no residual effects from the proceeding clear image. However, the blurred images differ in luminance thus setting a different baseline for the subsequent portrait. This is actually desired, so that the magnitude of the effect is not the result of a shift in overall luminance level (as occurs in Figure 2). Instead, each original and mirror-reversed blurred image sets the same baseline for their subsequent clear image. This is important since it is only the results of these two (original and mirror-reversed test-image) pupil diameters that will be compared against each other.

I manually recorded pleasantness scores for each face by taking verbal esthetic judgments using a 1–9 numerical scale, with one meaning most displeasing, five meaning neutral, and nine meaning most pleasing. Pleasingness is just one dimension of esthetics, but seemed to be appropriate based on a study that used five evaluative scales (e.g., pleasingness, likeability, preferability, interestingness, and complexity) (Russell and George, 1990). In Russell and George’s (1990) study, pleasingness was highly correlated with likeability and preferability, and was the highest in inter-subject agreement. The difference in verbal rating between the original and mirror-reverse images was then correlated with the difference between the average pupil diameters.

Following the stimulus presentation, observers were administered a questionnaire that pertained to their art training and their familiarity with the portraits. They were not told prior to the experimental session that they would see original and mirror-reversed images, but they may have become aware of this as the session progressed. To determine if observers noticed these mirror duplications, I asked whether they noticed anything unusual about the images at the end of the session. Sixteen of 40 observers reported noticing that a number of images were mirror-reversed. Only three observers had formal art training and nine reported that they had seen less than 25% of the portraits before. Thus, while many of these images are famous, most observers were not familiar with them.

## Results

### Pleasantness ratings

Before examining individual pleasantness ratings, I obtained each observer’s average pleasantness ratings for each of the eight portrait types (e.g., original right and left, mirror-reverse right and left, males and females). This resulted in each observer having eight data points. Then, pleasantness ratings for male and female portraits were submitted to a 2 (Portrait Gender: male vs. female) × 2 (Orientation: original vs. mirror-reversed) × 2 (Side of Face: left vs. right) repeated measures ANOVA. Males and female observers were included as a between-subjects factor, but showed no effect.

Figure 4 shows the means for each portrait group. There was a main effect for Side of Face with left-cheeks rated higher than right-cheeked individuals *F*(1, 39) = 11.55, *p* = 0.002, *d* = 1.07. Additionally, there were three significant interactions. First, there was a Side of Face by Orientation interaction *F*(1, 39) = 12.54, *p* = 0.001, *d* = 1.12. While left-side portraits (original and reversed) were rated higher than right portraits (see Side of Face main effect), right mirror reversals were rated higher than right originals (*M* = 4.23 vs. *M* = *4*.13), while left originals were rated higher than left mirror reversals (*M* = 4.75 vs. *M* = 4.57, respectively) [*t*(1, 78) = 2.56, *p* = 0.01, *d* = 0.59; *t*(1, 78) = 2.91, *p* = 0.005, *d* = 0.65]. That is, leftward appearing portraits, left originals and right reversals whose appearance to the observer seems to be left faced, are rated higher than right faced originals and left mirror-reversed (portraits viewed as seemingly right faced). Second, a Side of Face by Portrait Gender interaction was found *F*(1, 39) = 15.12, *p* = 0.001, *d* = 1.23 with left-side females portraits rated higher than right-side females portraits (*M* = 5.07 vs. *M* = 4.38), whereas the opposite is true for males portraits though to a lesser degree (*M* = 4.23 for left male portraits and *M* = 4.41 for right male portraits) [*t*(1, 78) = 3.22, *p* = 0.002, *d* = 0.72; *t*(1, 78) = 2.96, *p* = 0.004, *d* = 0.66]. Third, there was an Portrait Gender by Orientation *F*(1, 39) = 7.42 *p* = 0.097, *d* = 0.86, such that female mirror reversals were rated lower than original female portraits (*M* = 4.38 vs. *M* = 4.58), whereas the opposite relationship was found for males (*M* = 4.35 vs. *M* = 4.29) [*t*(1, 78) = 2.72, *p* = 0.008, *d* = 0.61; *t*(1, 78) = 2.51, *p* = 0.015, *d* = 0.015].

### Pupil size

**Figure 3:**
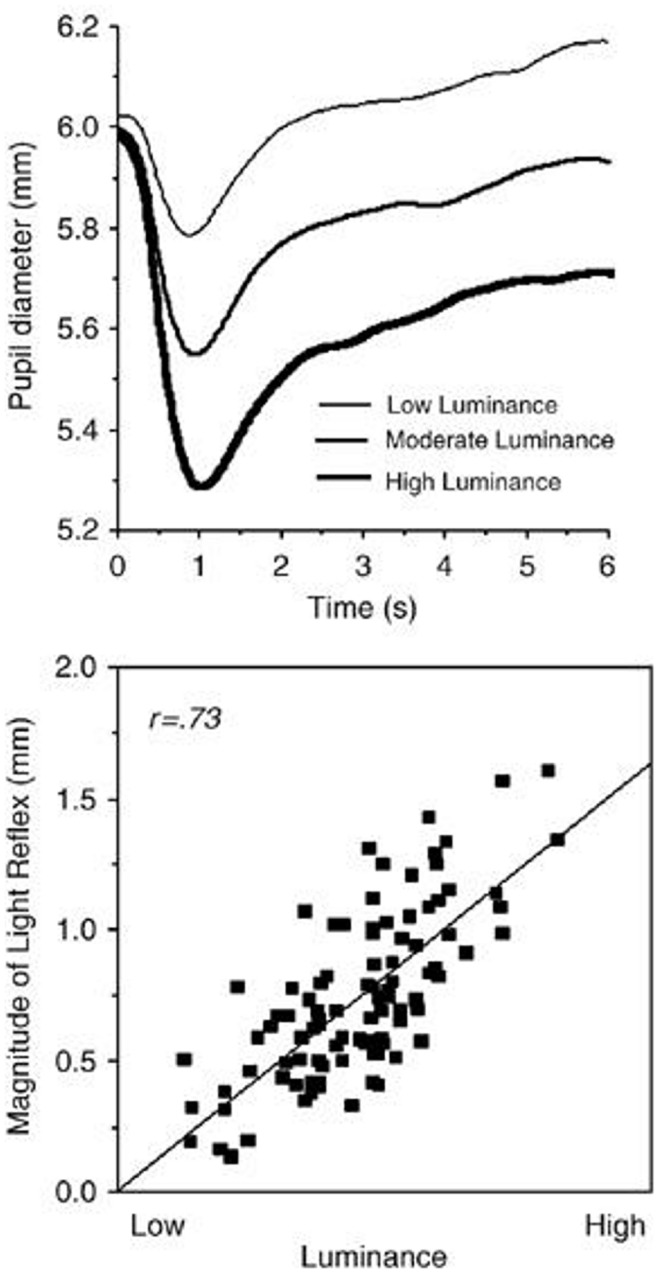
**(Top) Average pupil diameter over time as a function of luminance of the image viewed**. This variation is termed the light reflex. (Bottom) correlation of the magnitude of the light reflex as a function of image luminance (Taken from Bradley et al., 2008).

**Figure 4:**
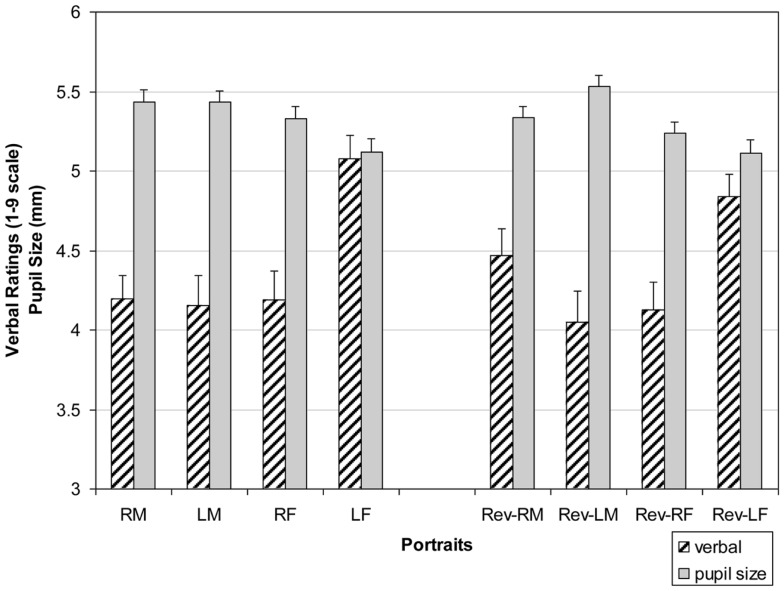
**Verbal ratings (striped bars) as a function of portrait type on a 1–9 scale, with 1 indicating most displeasing, 5 indicating neutral, and 9 indicating most pleasant**. Pupil size in mm (solid bars) as a function of portrait type (R = Right-cheeked, L = Left-cheeked, M = Males, F = Females, Rev = Mirror-reversed images). Error bars = SEM.

Before examining individual pupil size, I obtained each observer’s average pupil size for each of the eight types of portraits. Mean pupil sizes for these groups are shown in Figure 3 alongside the means of the previous three-way ANOVA conducted for verbal pleasingness. There was a main effect of portrait gender across the eight types of portraits *F*(1, 39) = 39.14, *p* < 0.0001, *d* = 1.98 (Figure 4). Average pupil diameter when viewing male portraits was larger (*M* = 5.5) than when viewing females portraits (*M* = 5.3) [*t*(1, 78) = 3.07, *p* = 0.003, *d* = 0.69]. This analysis also yielded a significant Side of Face × Orientation interaction *F*(1, 39) = 14.72, *p* = 0.001, *d* = 1.21. Original right-cheeked portraits (*M* = 5.42) elicited greater average pupil size than right-cheeked reversed portraits (*M* = 5.15) [*t*(1, 78) = 3.55, *p* = 0.001, *d* = 0.76]. Conversely, average pupil size was largest for left-cheeked reversed portraits (*M* = 5.44) than for original left-cheeked portraits (*M* = 5.23) [*t*(1, 78) = 3.13, *p* = 0.002, *d* = 0.72]. When these findings are examined alongside the Side of Face × Orientation interaction for verbal ratings, I find that images with the appearance of being right-cheeked yielded larger average pupil size and lower verbal ratings.

**Figure 5:**
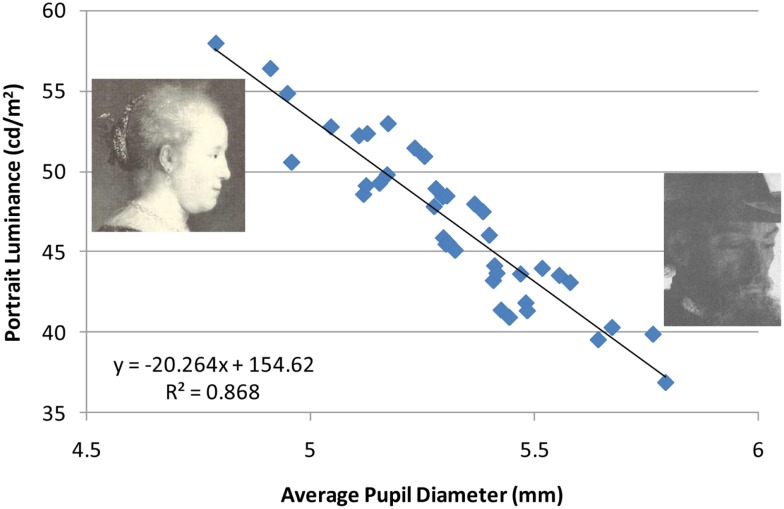
**Correlation between pupil diameter given the average luminance for each portrait (*n* = 40 observers)**. The figure includes the portraits with the highest and lowest average luminance.

Overall, pupil diameters were well within the normal range, where, as expected, the luminance of the portrait viewed dramatically affected pupil size (Figure 5; range = 4.71–5.82 mm). The fact that pupil contraction increased with an increase in image luminance reinforces my decision to use mirror-reversed images.

### Linear and quadratic relationships between pleasantness ratings and pupil diameter

**Figure 6:**
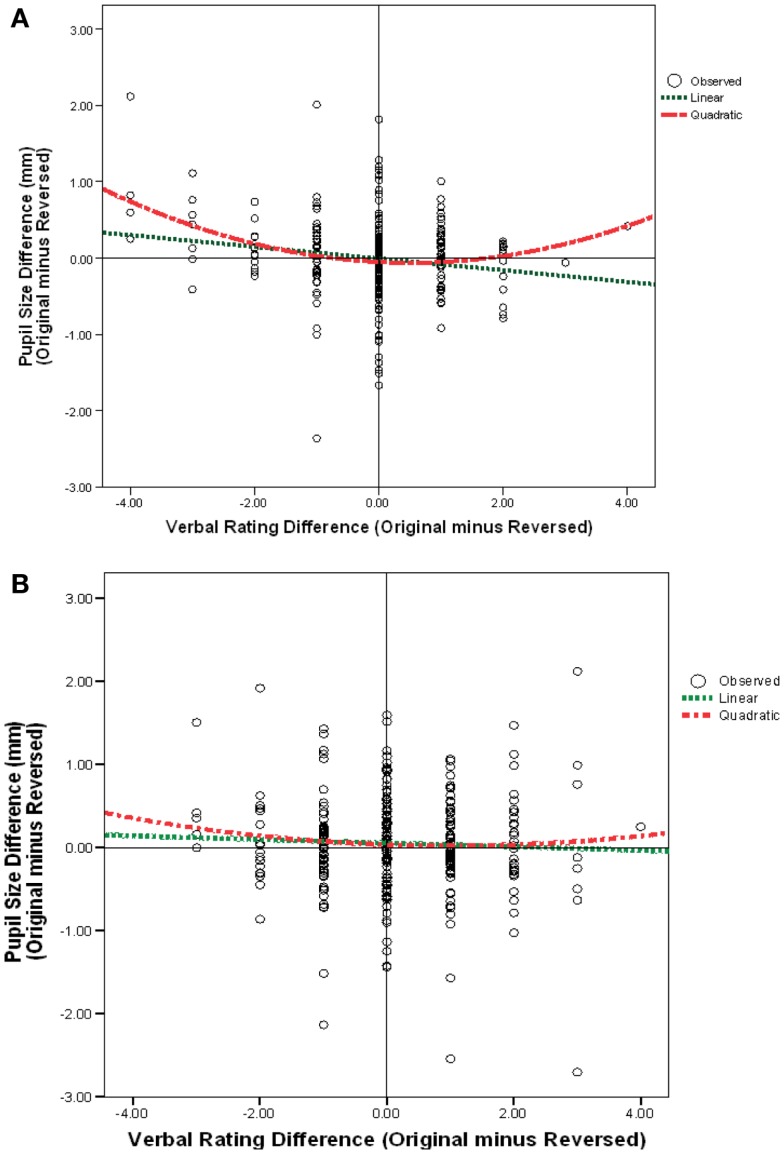
**Pupil size difference score (original-minus mirror-reversed) as a function of the difference in 1–9 verbal rating scores (between an original-minus mirror-reversed image) for all (A) male portraits, (B) female portraits**.

I examined linear and quadratic relationships between pleasantness and pupil size. First, a regression was computed to examine whether there was a linear relationship between pleasantness and pupil diameter. This was done by taking each portrait (original or mirror-reversed) for each observer as an individual case. Then difference scores between verbal pleasingness were regressed using the predictor variables of pupil size difference and quadric pupil size difference. Figure 6A shows that, for males, as the original verbal ratings became more positive (represented on the *x*-axis by verbal rating differences that were greater than zero), the original pupil size got smaller. Likewise, as original ratings became more negative (represented on the *x*-axis by verbal rating differences that were less than zero), the original pupil size got larger. This negative slope was statistically significant for male portraits *r*(39) = −0.41, *p* < 0.009, *d* = −0.90 (Figure 6A) but I failed to find a relationship for female portraits *r*(39) = −0.22, *p* = 0.178, *d* = −0.90 (Figure 6B). This means that when an original male portrait was preferred (whether right- or left-cheeked) the pupil was smaller while viewing the original portrait compared to the mirror-reversed image. Yet, when the mirror-reversed portrait was preferred (whether right- or left-cheeked) the pupil was smaller while viewing the mirror-reversed portrait compared to the original.

Next, linear and quadratic functions were entered into a regression model to evaluate if the relationship is better categorized by a quadratic function. The following quadratic regression model was computed separately for male and female portraits:

$$\mathrm{Pupil}=b_{0}+b_{1}\left(\mathrm{pleasantness}\right)+b_{2}\left(\mathrm{pleasantnes}s^{2}\right)$$

Figure 6A shows that there was a significant quadratic effect for male portraits *b* = 0.40, *t*(797) = 3.22, *p* = 0.002, *d* = 0.22 when the linear relationship was held constant. Pupil diameter was largest when there were the greatest differences in verbal ratings between an original and mirror-reversed male image. That is, when images were extremely liked or disliked, pupil size increased. This quadratic relationship accounted for significantly more of the variance than the linear relationship, *R*^2^ change = 0.32, *p* < 0.004, *d* = 0.22. However, for female portraits, the regression model failed to find a significant linear or quadratic relationship between pupil diameter and pleasantness *F*(2, 797) = 0.59, *p* = 0.32, *d* = 0.07 (Figure 6B). Pupil size could be confounded by the luminance of an image, so difference scores were calculated to compare across images. If difference scores were not used, and instead, pupil size for original and mirror-reversed images were used in the regression, then my findings would be seriously confounded because these values do not account for the changes in luminance/contrast across images and comparisons across images would be misleading. In sum, there were 800 total cases [i.e., 40 observers × 40 portraits (original-minus mirror-reversed)] used in the analysis.

$$\begin{array}{llll} \mathrm{Outcome}\;\left(\mathrm{DV}\right)\;\mathrm{variable} & =\mathrm{pupil}\;\mathrm{difference}\; & & \;\\ \mathrm{Predictor}\;\left(\mathrm{IVs}\right)\;\mathrm{variable} & =\mathrm{verbal}\;\mathrm{rating}\;\mathrm{difference;}\; & & \;\\ & \;\times \mathrm{quadratic}\;\mathrm{verbal}\;\mathrm{difference}\; & & \; \end{array}$$

It is important to realize that my images were not necessarily less pleasing in their non-original orientation. Instead, what I found was that only if there were a large difference in the rating between original and mirror-reversed images (where either orientation could have been the more pleasing image) there would also be a large difference in pupil size between those images.

**Figure 7:**
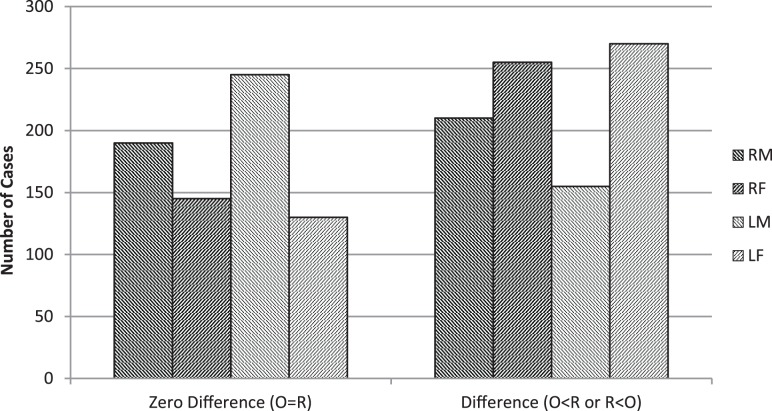
**Number of cases for all observers in which there was either a zero difference in verbal rating or there was a difference of any magnitude (with the original score being either more or less than the mirror-reversed image)**. (R = Right-cheeked, L = Left-cheeked, M = Males, F = Females).

As expected, there were almost as many zero differences between original and reversed image verbal ratings as there were difference scores (Figure 7). This inevitable outcome can potentially affect any of my linear and quadratic relationships.

## Discussion

While it has been shown that pupils get larger to intense (arousing) stimuli, I only replicated this for male portraits. Schirillo’s (2000) analysis of Rembrandt’s portraits suggests this may be because the perceived dominance of male portraits was rated higher than for female portraits. Dominance may be associated with positive (e.g., self-assurance, arrogance, and feeling bold or triumphant) or negative affective states (e.g., hostility, irritability, and anger) (Demaree et al., 2005). It is possible that while Rembrandt painted males to exhibit these positive dominant traits (see Humphrey and McManus, 1973), negative dominance traits may have also been captured. Thus, the negative linear relationship between pleasantness ratings and pupil diameter for males is consistent with Tinio and Robertson (1969), who found that aggressive Thematic Apperception Test cards elicited larger pupil size than control cards. This implies that Rembrandt’s male portraits may actually be perceived as domineering which is consistent with Libby et al. (1973) and Woodmansee (1967) who found that unpleasant images were associated with larger pupil sizes compared to pleasant images.

Given that my verbal ratings of pleasingness are a self-report measure of what may be an emotive expression, it was of interest to determine their relationship with an unconscious indicator of pleasingness (i.e., pupil diameter) as suggested by Hess and others, and how this in turn might be related to the emotional content of the facial musculature displayed in the images. For example, self-report assumptions regarding dominance can be present in the emotional qualities of the portraits, which may be captured by pupil size relationships. I have explored these variables while simultaneously eliminating Stern and Strock’s (1987) concern that one drawback to pupillometry is that changes associated with such variables are considerably smaller than those associated with illumination effects.

This new methodology eliminates Hess’ (1972) fluctuations in luminance and contrast across images allowing us to observe how pupil size varies as a function of the differences in verbal reports of pleasant and less pleasant original and mirror-reversed portraits. This was done by *only* comparing measurements between original and mirror-reversed images which makes image contrast and luminance irrelevant. This also means I do not require a baseline pupil size from the blurred images; since pupil size should not vary between original and mirror-reversed images because such images do not vary in image contrast and luminance. If they do differ, this must be because the emotional content of the images vary, not their image contrast or luminance.

Given that it is impossible to equate apparent contrast or mean luminance across images, research testing Hess’ hypothesis had ceased. However, my improved methodology does not need to equate apparent contrast or mean luminance since this automatically occurs by comparing pupil size only across an original and its mirror-reversed image. In essence, I created a methodology that reexamined the relationship between a self-report and emotive measure (i.e., pleasingness) and an unconscious physiological measure (i.e., pupil size). I show that Hess (1965, 1972) was incorrect by focusing on valence. Instead he should have focused on arousal, since arousal drives the effect for male portraits not valence.

Male portraits showed both a linear and quadratic relationship between pupil diameter and esthetic judgments of pleasantness. The linear model indicates that pupil diameter increased when viewing negative male portraits and decreased when viewing positive male portraits, whereas a quadratic model shows that pupil diameter increases to both highly pleasant and unpleasant male portraits. Thus, it is plausible that researchers who used only a linear function found that unpleasant images were associated with larger pupil sizes compared to pleasant images (Woodmansee, 1967; Tinio and Robertson, 1969; Libby et al., 1973). These findings do not support Hess’ (1965) prediction, which suggested that unpleasant images would have been associated with a smaller pupil diameter.

However, a quadratic relationship accounts for significantly more variance than a linear relationship. In this case, pupil size increased with large differences among pleasantness ratings for male faces. This is consistent with previous findings that pupil size is related to stimulus intensity rather than their specific positive or negative content (Janisse, 1973, 1974, 1977; Aboyoun and Dabbs, 1998). For example, arousing situations have been shown to produce larger pupil diameter in non-visual stimuli studies. Nunnally et al. (1967) found that painfully loud sounds increased muscle tension causing larger pupil size. They also found an increase in pupil diameter when observers expected to hear a gunshot. Likewise, Polt (1970) found larger pupil size during mental arithmetic tasks when observers believed they would be shocked for incorrect answers. This may also account for why females failed to show an effect of arousal. I suggest that females are considered pleasing (not aversive) compared to males, thus when viewing female portraits one’s pupil size is already approaching the floor, and thereby has less room to show arousal effects.

The esthetic verbal pleasantness judgments suggest that the observers were attending to the actual facial physiognomy of the posers and that pleasantness was determined to a lesser degree by the orientation the portrait faced. In agreement with prior research (Schirillo and Fox, 2006), the left-side of women’s faces were rated as more esthetically pleasant than their right-side. This suggests that for women it is important to express more emotive facial qualities than males, in agreement with Nicholls et al. (1999) and the right-hemisphere model of emotion lateralization. Yet how emotion may be lateralized in the cerebral hemispheres is still under debate (Davidson, 1995; Demaree et al., 2005; Killgore and Yurgelun-Todd, 2007). One recent argument is that an approach/withdrawal model may provide a more appropriate fit to the data as opposed to the positive/negative hemispheric difference model (Demaree et al., 2005). Davidson and others interpretation of the left/right differences in emotion valence is that the right cerebral hemisphere regulates withdrawal behaviors whereas the left cerebral hemisphere regulates approach behaviors (Kinsbourne, 1982; Davidson, 1984, 1992, 1995; Davidson et al., 1990; Fox, 1991). If this is the case, as stated in Schirillo (2000), I speculate that Rembrandt preferred to paint females left-side because it captured the attractive quality of being demure.

Prehn et al. (2013) demonstrated that oxytocin increased saliency of all social stimuli regardless whether faces were male or female. In the placebo group (without oxytocin treatment), however, they found a strong gender effect (which decreased after oxytocin treatment). However, they used Winmorph 3.01 (www.debugmode.com/winmorph) to transform neutral expressions into all of the emotional ones in 5% steps. My study had no such control. Interestingly, their placebo “happy” male and female faces did not significantly differ in recorded intensity levels, nor recorded pupil size. This would have been the condition that I most like replicated. Like me, they found increased pupil dilations during the processing of male compared with female faces, which they attribute to reflecting men’s (they only used male subjects) lower interest in male faces. However, I used both sexes as subjects and found no significant differences between groups. Yet their oxytocin manipulation is evidence that increased pupillary responses have been observed when stimuli are emotionally salient. But I controlled for saliency as much as possible by matching for valence and dominance, so I do not believe this was a factor in my experiment.

In summary, this study provides a new methodology to research the association between pupil diameter and esthetic verbal judgments. Based on its findings, Hess’ (1965, 1972) hypothesis that pupils dilate to pleasant images and constrict to displeasing images seems incorrect. Instead, at least for male portraits, pupil size is a function of arousal such that pupil size difference increases when the difference in verbal reports are both most pleasant and most displeasing. I consider the possibility that this is related to perceived dominance (Ellis, 2006), in that a linear function showed that faces rated low in esthetic pleasantness evoked the largest pupil diameter. I consider the possibility that this is related to disliking perceived threat (Darwin, 1872), which may have been a dominance trait that Rembrandt inadvertently depicted.

## Conflict of Interest Statement

The author declares that the research was conducted in the absence of any commercial or financial relationships that could be construed as a potential conflict of interest.

## Appendix

### Listing of painting titles (taken from Bredius, 1969).

#### Left facing females

1. Rembrandt’s Sister, 1632. Stockholm, National Museum (Br. 85)
2. Saskia. 1643. Cassel, Gemaldegalerie (Br. 101).
3. Saskia. 1636. Hartford, Conn., Wadsworth Atheneum (Br. 105).
4. Hendrickje Stoffels as Flora. 1654. New York, Metropolitain of Art (Br. 114).
5. Amalia van Solms. 1632. Paris, Musee Jacquemart-Andre (Br. 99).

#### Right facing females

1. Rembrandt’s Mother as a Biblical Prophetess (Hannah?). 1631. Amsterdam, Rijksmuseum (Br. 69).
2. Saskia. 1633. Washington, National Gallery of Art (Widener Collection) (Br. 96).
3. Rembrandt’s Sister. 1634. Indianapolis, John Herron Art Museum (Br. 100).
4. A Woman in Fanciful Costume. 1648. Baltimore, The Walters Art Gallery (Br. 386).
5. A Young Girl Seated. 1660. London, E. S. Borthwick Norton Sale, May 15. 1953 (Br. 393).

#### Left facing males

1. Head of an Old Man. Copenhagen, Statens Museum (Br. 136).
2. A Man in Oriental Costume. 1633. Munich, Alte Pinakothek (Br. 178).
3. A Bearded Man. 1646. Cassel, Germaldegalerie (Br. 230).
4. Study of an Old Man. 1640. Detroit, Mrs. Standish Backus (Br. 244).
5. Portrait of an Old Man in a Pearl-Trimmed Hat. 1662. Dresden, Gemaldegalirie (Br. 324).

#### Right facing males

1. Self-Portrait. 1629. Munich, Alte Pinakothek (Br. 2).
2. Self-Portrait. Not dated. The Hague, Mauritshuis (Br. 24).
3. An Old Man with a Gold Chain. 1630. Los Angeles, Hans Cohn (Br. 149).
4. Portrait of a Man Reading. 1645. Williamstown, Mass., The Sterling and Francine Clark Art Museum (Br. 238).
5. Study of the Head of an Old Man. 1661. New York, John Hay Whitney (Br. 261).

## References

[B1] Aboyoun D. C. Dabbs J. M. (1998). The Hess pupil dilation findings: sex or novelty? Soc. Behav. Pers. 26, 415–420 10.2224/sbp.1998.26.4.415

[B2] Ahern G. L. Schwartz G. E. (1979). Differential lateralization for positive versus negative emotion. Neuropsychologia 17, 693–698 10.1016/0028-3932(79)90045-9 522984

[B3] Annett M. Kilshaw D. (1983). Right and left-hand skill. 1. Estimating the parameters of the distribution of L-R differences in males and females. Br. J. Psychol. 74, 269–283 10.1111/j.2044-8295.1983.tb01862.x 6871579

[B4] Bachman T. (2007). When beauty breaks down: investigation of the effect of spatial quantisation on aesthetic evaluation of facial images. Perception 36, 840–849 10.1068/p5509 17718363

[B5] Beatty J. Lucero-Wagoner B. (2000). “The pupillary system,” in Handbook of Psychophysiology, eds Cacioppo J. T. Tassinary L. G. Berntson G. G. (Cambridge: Cambridge University Press), 14–162

[B6] Borod J. C. Caron H. S. (1980). Facedness and emotion related to lateral dominance, sex and expression type. Neuropsychologia 18, 237–241 10.1016/0028-3932(80)90070-6 7383316

[B7] Borod J. C. Haywood C. S. Koff E. (1997). Neuropsychological aspects of facial asymmetry during emotional expression: a review of the normal adult literature. Neuropsychol. Rev. 7, 41–60 10.1007/BF02876972 9243530

[B8] Borod J. C. Kent J. Koff E. Martin C. Alpert M. (1988). Facial asymmetry while posing positive and negative emotions: support for the right hemisphere hypothesis. Neuropsychologia 26, 759–764 10.1016/0028-3932(88)90013-9 3211295

[B9] Bradley M. M. Miccoli L. Escrig M. A. Lang P. (2008). The pupil as a measure of emotional arousal and autonomic activation. Psychophysiology 45, 602–607 10.1111/j.1469-8986.2008.00654.x 18282202 PMC3612940

[B10] Brodal A. (1965). The Cranial Nerves. Oxford: Blackwell

[B11] Bryden M. P. Ley R. G. (1983). “Right-hemispheric involvement in the perception and expression of emotion in normal humans,” in Neuropsychology of Human Emotion, eds Heilman K. M. Satz P. (New York: Guilford Press), 6–44

[B12] Conesa J. Brunold-Conesa C. Miron M. (1995). Incidence of the half-left profile pose in single-subject portraits. Percept. Mot. Skills 81, 920–922 10.2466/pms.1995.81.3.920 8668453

[B13] Darwin C. (1872). The Expression of the Emotions in Man and Animals. New York: D. Appleton and Co

[B14] Davidson R. J. (1984). “Affect, cognition, and hemispheric specialization,” in Emotions, Cognition, and Behavior, eds Izard C. E. I. Kagan J. Zajonc R. B. (Cambridge, MA: Cambridge University Press), 320–365

[B15] Davidson R. J. (1992). Emotion and affective style: hemispheric substrates. Psychol. Sci. 3, 39–43 10.1111/j.1467-9280.1992.tb00254.x

[B16] Davidson R. J. (1995). “Cerebral asymmetry, emotion, and affective style,” in Brain Asymmetry, eds Davidson R. J. Hughdahl K. (Cambridge, MA: MIT Press), 361–388

[B17] Davidson R. J. Ekman P. Saron C. D. Senuluis J. A. Friesen W. V. (1990). Approach-withdrawal and cerebral asymmetry: emotional expression and brain physiology I. J. Pers. Soc. Psychol. 58, 330–341 10.1037/0022-3514.58.2.330 2319445

[B18] Demaree H. A. Everhart D. E. Youngstrom E. A. Harrison D. W. (2005). Brain lateralization of emotional processing: historical roots and a future incorporating “dominance.” Behav. Cogn. Neurosci. Rev. 4, 3–20 10.1177/1534582305276837 15886400

[B19] Dunbar N. E. Burgoon J. K. (2005). Perceptions of power and interactional dominance in interpersonal relationships. J. Soc. Pers. Relat. 22, 207–233 10.1177/02654075050509448856951

[B20] Ellis L. (2006). Gender differences in smiling: an evolutionary neuroandrogenic theory. Physiol. Behav. 88, 303–308 10.1016/j.physbeh.2006.03.034 16753190

[B21] Fitzgerald H. E. (1968). Autonomic pupillary reflex activity during early infancy and its relation to social and non-social and non-social visual stimuli. Diss. Abstr. 28, 3896B–3897B 10.1016/0022-0965(68)90127-6 5687128

[B22] Fox N. A. (1991). If it’s not left, it’s right: electroencephalograph asymmetry and the development of emotion. Am. Psychol. 46, 863–872 10.1037/0003-066X.46.8.863 1928939

[B23] Fridlund A. J. Izard C. (1983). “Electromyographic studies of facial expressions of emotions and patterns of emotion,” in Social Psychophysiology: A Sourcebook, eds Cacioppo J. T. Petty R. E. (New York: Guilford Press), 254–261

[B24] Gainotti G. (1969). “Catastrophic” reactions and manifestations of indifferences during cerebral disorders. Neuropsychologia 7, 195–204 10.1016/0028-3932(69)90017-71288661

[B25] Gainotti G. (1972). Emotional behavior and hemispheric side of lesion. Cortex 8, 41–55 10.1016/S0010-9452(72)80026-1 5031258

[B26] Goldwater B. C. (1972). Psychological significance of pupillary movements. Psychol. Bull. 77, 340–355 10.1037/h0032456 5021049

[B27] Gordon I. (1974). “Left and right in art,” in Psychology and the Arts, ed. O’Hare D. (Brighton: Harevesor), 211–241

[B28] Grüsser O. J. Selke T. Zynda B. (1988). “Cerebral lateralization and some implications for art, aesthetic perception, and artistic creativity,” in Beauty & The Brain: Biological Aspects of Aesthetics, eds Reutshcler I. Herzberger B. Epstein D. (Boston: Berkhäuer Verlag), 257–293

[B29] Hess E. H. (1965). Attitude and pupil size. Sci. Am. 212, 46–54 10.1038/scientificamerican0465-46 14261525

[B30] Hess E. H. (1972). “Pupillometrics: a method of studying mental, emotional, and sensory processes,” in Handbook of Psychophysiology, eds Greenfield N. S. Sternbach R. A. (New York: Holt, Rinehart & Winston), 491–531

[B31] Hess E. H. Polt J. M. (1960). Pupil size as related to interest value of visual stimuli. Science 132, 349–350 10.1126/science.132.3423.349 14401489

[B32] Hess E. H. Seltzer A. L. Shlien J. M. (1965). Pupil response of hetero- and homosexual males to pictures of men and women: a pilot study. J. Abnorm. Psychol. 70, 165–168 10.1037/h0021978 14297654

[B33] Humphrey N. McManus C. (1973). Status and the left cheek. New Sci. 23, 437–439

[B34] Janisse M. P. (1973). Pupil size and affect: a critical review of the literature since 1960. Can. Psychol. 14, 311–329 10.1037/h0082230

[B35] Janisse M. P. (1974). Pupil size, affect and exposure frequency. Soc. Behav. Pers. 2, 125–146 10.2224/sbp.1974.2.2.12512122136

[B36] Janisse M. P. (1977). Pupillometry: The Psychology of the Pupillary Response. New York, NY: John Wiley & Sons

[B37] Jasari A. Tranel D. Adolphs R. (2000). A valence-specific lateral bias for discriminating emotional facial expressions in free field. Cogn. Emot. 14, 341–353 10.1080/026999300378860

[B38] Killgore W. D. S. Yurgelun-Todd D. A. (2007). The right-hemisphere and valence hypotheses: could they both be right (and sometimes left)? Soc. Cogn. Affect. Neurosci. 2, 240–250 10.1093/scan/nsm020 18985144 PMC2569811

[B39] Kinsbourne M. (1982). Hemispheric specialization and the growth of human understanding. Am. Psychol. 37, 411–420 10.1037/0003-066X.37.4.411 7103239

[B40] Kohn M. Clynes M. (1969). Color dynamics of the pupil. Ann. N. Y. Acad. Sci. 156, 931–950 10.1111/j.1749-6632.1969.tb14024.x 5258025

[B41] Libby W. L. Lacey B. C. Lacey J. I. (1973). Pupillary and cardiac activity during visual attention. Psychophysiology 10, 270–294 10.1111/j.1469-8986.1973.tb00526.x 4702521

[B42] Lindell A. K. (2013). Continuities in emotion lateralization in human and non-human primates. Front. Hum. Neurosci. 7:464 10.3389/fnhum.2013.00464 23964230 PMC3737467

[B43] Locher P. L. (1996). The contribution of eye-movement research to an understanding of pictorial balance perception: a review of the literature. Empiric. Stud. Arts 14, 143–163 10.2190/D77M-3NU4-DQ88-H1QG

[B44] Loewenfeld I. E. (1966). Comment on Hess’ findings. Surv. Ophthalmol. 11, 293–294

[B45] Loewenfeld I. E. (1999). The Pupil: Anatomy, Physiology, and Clinical Applications. Boston: Butterworth-Heinemann

[B46] Loftus G. R. (1985). Picture perception: effects of luminance on available information and information-extraction rate. J. Exp. Psychol. Gen. 114, 342–356 10.1037/0096-3445.114.3.342 3161980

[B47] Mandal M. K. Tandon S. C. Asthana H. S. (1991). Right brain damage impairs recognition of negative emotions. Cortex 27, 247–253 10.1016/S0010-9452(13)80129-3 1879153

[B48] Mannan S. Ruddock K. H. Wooding D. S. (1995). Automatic control of saccadic eye movements made in visual inspection of briefly presented 2-D images. Spat. Vis. 9, 363–386 10.1163/156856895X00052 8962841

[B49] McManus C. Humphrey N. (1973). Turning the left cheek. Nature 243, 271–272 10.1038/243271a0

[B50] Miller R. L. (1967). The Clinical Validation of the Pupillary Response: The Effect of Chromatic and Achromatic Stimuli Upon Pupil Responsivity. Doctoral Dissertation, Michigan State University, East Lansing

[B51] Nakayama M. (2006). “Influence of blink on pupillary indices,” in Biomedical Circuits and Systems Conference (London: IEEE BioCAS 2006), 29–32

[B52] Natale M. Gur R. E. Gur R. C. (1983). Hemispheric asymmetries in processing emotional expressions. Neuropsychologia 21, 555–565 10.1016/0028-3932(83)90011-8 6646407

[B53] Nicholls M. E. R. Clode D. Lindell A. K. Wood A. G. (2002a). Which cheek to turn? The effect of gender and emotional expressivity on posing behavior. Brain Cogn. 48, 480–484 10.1006/brcg.2001.1402 12030492

[B54] Nicholls M. E. R. Wolfgang B. J. Clode D. Lindell A. K. (2002b). The effect of left and right poses on the expression of facial emotion. Neuropsychologia 40, 1662–1665 10.1016/S0028-3932(02)00024-6 11992654

[B55] Nicholls M. E. R. Clode D. Wood S. J. Wood A. G. (1999). Laterality of expression in portraiture: putting your best cheek forward. Proc. R. Soc. Lond. 226, 1517–1522 10.1098/rspb.1999.0809 10467743 PMC1690171

[B56] Nicholls M. E. R. Ellis B. E. Clement J. G. Yoshino M. (2000). Detecting hemifacial asymmetries in emotional expression with three-dimensional computerized image analysis. Proc. R. Soc. Lond. Biol. Sci. 271, 663–668 10.1098/rspb.2003.2660 15209097 PMC1691649

[B57] Nunnally J. C. Knott P. D. Duchnowski A. Parker R. (1967). Pupillary responses as a general measure of activation. Percept. Psychophys. 2, 149–155

[B58] Polt J. M. (1970). Effect of threat of shock on pupillary response in a problem-solving task. Percept. Mot. Skills 31, 587–593 10.2466/pms.1970.31.2.587 5492337

[B59] Powell W. R. Schirillo J. A. (2009). Asymmetrical facial expressions in portraits and hemispheric laterality: a literature review. Laterality 14, 545–572 10.1080/13576500802680336 19214864

[B60] Prehn K. Kazzer P. Lischke A. Heinrichs M. Herpertz S. C. Domes G. (2013). Effects of intranasal oxytocin on pupil dilation indicate increased salience of socioaffective stimuli. Psychophysiology 50, 528–537 10.1111/psyp.12042 23551070

[B61] Richer F. Silverman C. Beatty J. (1983). Response selection and initiation in speeded reactions: a pupillometric analysis. J. Exp. Psychol. Hum. Percept. Perform. 3, 360–370 6223976 10.1037//0096-1523.9.3.360

[B62] Rinn W. E. (1984). The neuropsychology of facial expression: a review of the neurological and psychological mechanisms for producing facial expressions. Psychol. Bull. 95, 52–77 10.1037/0033-2909.95.1.52 6242437

[B63] Rossi G. F. Rosadini G. (1967). “Experimental analysis of cerebral dominance in man,” in Brain Mechanisms Underlying Speech and Language, eds Millikan C. H. Darley F. L. (New York: Grune and Stratton), 167–189

[B64] Russell P. A. George D. A. (1990). Relationships between aesthetic response scales applied to paintings. Empiric. Stud. Arts 8, 15–30 10.2190/AU1R-6UXE-T14R-04WQ

[B65] Sackeim H. A. Greenberg M. S. Weiman A. L. Gurr R. C. (1982). Hemispheric asymmetry in the expression of positive and negative emotions. Arch. Neurol. 39, 210–218 10.1001/archneur.1982.00510160016003 7041863

[B66] Sackeim H. A. Gur R. C. (1978). Lateral asymmetry in intensity of emotional expression. Neuropsychologia 16, 473–481 10.1016/0028-3932(78)90070-2 692859

[B67] Sackeim H. A. Gur R. C. (1983). “Facial asymmetry and the communication of emotion,” in Social Psychophysiology, eds Cacioppo J. T. Petty R. E. (New York: Guilford), 307–352

[B68] Sackeim H. A. Gur R. C. Saucy M. C. (1978). Emotions are expressed more intensely on the left side of the face. Science 202, 434–436 10.1126/science.705335 705335

[B69] Schiff B. B. Lamon M. (1989). Inducing emotion by unilateral contraction of facial muscles: a new look at hemispheric specialization and the experience of emotion. Neuropsychologia 27, 923–935 10.1016/0028-3932(89)90068-7 2771031

[B70] Schirillo J. Fox M. (2006). Rembrandt’s portraits: approach or avoid? Leonardo 39, 253–256 10.1162/leon.2006.39.3.253

[B71] Schirillo J. A. (2000). Hemispheric asymmetries and gender influence Rembrandt’s portrait orientations. Neuropsychologia 38, 1593–1606 10.1016/S0028-3932(00)00063-4 11074082

[B72] Schwartz G. E. Ahern G. L. Brown S. (1979). Lateralized facial muscle response to positive and negative emotional stimuli. Psychophysiology 16, 561–571 10.1111/j.1469-8986.1979.tb01521.x 515297

[B73] Silberman E. K. Weingartner H. (1986). Hemispheric lateralization of functions related to emotion. Brain Cogn. 5, 322–353 10.1016/0278-2626(86)90035-7 3530287

[B74] Simms T. M. (1967). Pupillary response of male and female subjects to pupillary difference in male and female picture stimuli. Percept. Psychophys. 2, 553–555

[B75] Smith J. K. Smith L. F. (2001). Spending time on art. Empiric. Stud. Arts 19, 229–236 10.2190/5MQM-59JH-X21R-JN5J

[B76] Steinhauer S. R. Siegle G. J. Condray R. Pless M. (2004). Sympathetic and parasympathetic innervation of pupillary dilation during sustained processing. Int. J. Psychophysiol. 52, 77–86 10.1016/j.ijpsycho.2003.12.005 15003374

[B77] Stern J. A. Strock B. D. (1987). “Oculomotor activity and user-system interaction in the workplace,” in Psychophysiology and the Electronic Workplace, eds Gale A. Christie B. (Oxford: John Wiley & Sons), 239–254

[B78] Tinio F. Robertson M. (1969). Examination of two indices of hostility: fantasy and change in pupil size. Proc. Annu. Convent. Am. Psychol. Assoc. 4, 173–174

[B79] Woodmansee J. J. (1966). “Methodological problems in pupillographic experiments,” in Proceedings of the Annual Convention of the American Psychological Association, 133–134

[B80] Woodmansee J. J. (1967). The pupil reaction as an index of positive and negative affect. Paper Presented at the Convention of the American Psychological Association, Washington, DC

